# Biological Safety of Camel Milk After Albendazole and Ivermectin Treatment

**DOI:** 10.3390/vetsci12121178

**Published:** 2025-12-10

**Authors:** Gaukhar Konuspayeva, Zauresh Bilal, Nurlan Akhmetsadykov, Shynar Akhmetsadykova, Zhaidar Musyaev, Farida Amutova, Zaira Kabdullina, Dariga Utemuratova, Bernard Faye

**Affiliations:** 1 Biotechnology Department, Al-Farabi Kazakh National University, 71 Al-Farabi Avenue, Almaty 050040, Kazakhstan; konuspayevags@hotmail.fr (G.K.); bilalzauresh@gmail.com (Z.B.); amutovafb@gmail.com (F.A.); zaira01211@gmail.com (Z.K.); utemuratovadariga@gmail.com (D.U.); 2 LLP “Scientific and Production Enterprise Antigen”, 4, Azerbayeva Str., Almaty 040905, Kazakhstan; nurlan.akhmetsadykov@gmail.com (N.A.); shynar.akhmetsadykova@gmail.com (S.A.); antigen.chem@gmail.com (Z.M.); 3 LLP “Kazakh Research Institute for Livestock and Fodder Production”, Almaty 050035, Kazakhstan; 4 Center of International Cooperation on Agriculture Research for Development–CIRAD, UMR SELMET, Campus International de Baillarguet, 34398 Montpellier, France

**Keywords:** camel milk, antiparasitic treatment, Albendazole, Ivermectin, biosafety

## Abstract

The problem of Albendazole and Ivermectin medicine residues in milk is a current question in food safety. With no data being available, the present study aimed to assess the risk of antiparasitic residues in camel milk after injection at curative dose. For the experiment, 10 camels were allocated to two groups, treated with Albendazole and Ivermectin, respectively; milk samples were taken every day for the first 4 days, then approximately every 2 weeks for 6 months. In addition, blood samples were taken for the Albendazole group. The samples were analysed by HPLC. On average, for Albendazole, the maximum concentration appeared on day 2 (52 ± 65.2 µg/mL) and day 48 (50 ± 63.1 µg/mL). After 210 days, the residues remained at around 11 µg/mL (MRL = 0.1 µg/mL). The kinetic assessment in the blood samples showed a slight shift in relation to that of milk during the first days of the experiment, but there was no significant difference between them. For Ivermectin, only one peak appeared on day 101 (0,96 ± 1,19 µg/mL), and after 172 days, the mean concentration was still 0.12 µg/mL (MRL = 0.01 µg/mL). The persistence of the residues for a long time after injection leads us to consider that specific guidelines should be proposed for ensuring the safety of camel milk.

## Simple Summary

To avoid medicine residues in milk, a withdrawal period is generally applied. However, the application of this rule for milk after the antiparasitic treatment of camels is generally based on recommendations regarding cattle, despite the fact that pharmacokinetic excretion in camel milk could deeply differ from that of cow milk. The present study aims to assess Albendazole and Ivermectin residues, two antiparasitics widely used in veterinary practice, in camel milk until almost 6 months after injection. The results showed important residues in some animals even after 48 days for Albendazole (Maximum Residue Level—MRL = 0.1 µg/mL) and up to 3 months for Ivermectin (MRL = 0.01 µg/mL), leading us to propose a specific withdrawal period for this species.

## 1. Introduction

Parasitic infections are among the main factors limiting the productivity, health, and welfare of Old World camels, particularly in regions where extensive or semi-extensive husbandry is practiced. Gastrointestinal nematodes, cestodes, trematodes, and ectoparasites are commonly found in both dromedaries and Bactrian camels across Central Asia, including Kazakhstan [1]. Despite the increasing economic importance of camel milk and meat production, only a few veterinary antiparasitic drugs are officially registered for use in camels. As a result, treatments are often adapted from protocols developed for cattle or small ruminants, because species-specific pharmacokinetic and safety data for camels remain limited [2]. Indeed, there is a lack of data regarding the residual risks in camel milk. Yet, it is known that the pharmacokinetics of these antiparasitics depend on the animal species [3].

Albendazole (ALB) and Ivermectin (IVM) are widely used worldwide for controlling internal or external parasites in farm animals, including camels [4,5,6,7]. Albendazole, a benzimidazole derivative, shows broad-spectrum activity against gastrointestinal nematodes, cestodes, and some trematodes. Ivermectin, a macrocyclic lactone, is commonly applied for controlling both internal and external parasites. However, international guidelines strictly regulate the use of these drugs in food-producing animals due to the risk of residues in milk and meat. Albendazole is generally contraindicated in lactating animals used to produce milk for human consumption, except where withdrawal periods are clearly defined; meanwhile, Ivermectin’s use in lactating animals depends on the formulation, route of administration, and national registration [8]. Very few studies are available on Ivermectin or Albendazole residues in camel milk [9]. For example, Bengoumi et al. [10] have investigated the pharmacokinetics of Eprinomectin, an antiparasitic drug derived from Avermectin, in camel plasma and milk. To our knowledge, there are no published studies on Albendazole residue in camel milk; such studies do exist for milk from other species, such as goat [11], sheep, and cattle [12]. At the same time, camel milk is increasingly present on the market and consumed outside traditional nomad camps [13,14]. Moreover, with the development of more intensive dairy camel farming, antiparasitic prevention should be more systematic due to the higher risk of residues being present in camel products.

In Kazakhstan, veterinary drug regulations are in line with the Eurasian Economic Union (EAEU) legislation [15]. Injectable Ivermectin formulations are not registered for use in lactating dairy cattle, and similar restrictions are applied to other milk-producing species, including camels, because maximum residue limits (MRLs) for camel milk have not been officially established. Albendazole is also strictly limited during lactation, and its use is typically allowed only in non-lactating animals or with extended withdrawal periods [15]. Despite these regulations, some farmers continue to administer these drugs to lactating camels, creating potential risks of drug residues in milk and raising public health concerns.

These regulatory constraints, combined with the rapid development of the camel dairy sector in Kazakhstan, present practical challenges for herd health management. Producers often rely on off-label use, empirical dosing, or careful timing of treatments to control parasitic infections. At the same time, recent studies highlight the need for species-specific pharmacological information, as camels differ physiologically and metabolically from other ruminants, which may affect drug absorption, distribution, metabolism, and residue depletion [1,2].

Therefore, this article aims to assess the risk of antiparasitic residues in camel milk above the MRL for consumer health and to provide practical recommendations for their application in camel husbandry, with particular focus on Kazakhstan [8].

## 2. Materials and Methods

### 2.1. Animals

A total of ten one-humped lactating camels (*Camelus dromedarius*, Aruana breed, dairy type), aged 6–7 years, 5–6 months postpartum, were included in the study. The weight of each camel varies from 460 to 480 kg and their milk production was quite comparable (around 4 L/day at the beginning of the trial and 3 L at the end). The animals were divided into two treatment groups, with five camels receiving Albendazole (Ricobendazole) and five camels receiving Ivermectin, according to manufacturer instructions. All animals were clinically healthy at the beginning of the trial, although minor health fluctuations during the extended sampling period could not be excluded.

The camels were maintained under identical farm management conditions at Otemis-Ata farm (Akshi village, Yili district, Almaty region, Kazakhstan). In this region, the dromedary camel is the predominant dairy animal, and camel milk represents the main source of milk for consumers. They had continuous access to natural pasture supplemented with hay, and water was available ad libitum throughout the study period (Milking was performed manually twice daily during the autumn–winter months and three times daily in the spring–summer season, following local dairy practices. Individual daily milk yields were not recorded, as the focus was on milk quality and residue kinetics rather than productivity. 

All experimental procedures were conducted in accordance with national guidelines for animal welfare, and the protocol complied with ethical standards for research on food-producing animals. The trial was achieved in accordance with ethics committee guidelines of Co Antigen LTD (protocol 11/1, from 4 July 2023) and based on the order of the Minister of Health of the Republic of Kazakhstan dated 19 November 2009, No. 744. It started on 28 August 2023, and ended on 19 February 2024. A post-trial follow-up measurement was performed on 3 July 2024 to test the stability of the former results, but as most of the camels having stopped lactating at this date, the results were not included. Thus, the total duration of the trial was six months. This period was also chosen because antiparasitic treatments are usually administered at the end of summer.

### 2.2. Drugs and Treatment

#### 2.2.1. Anthelmintic Drug

The preparation used was Ricazole^®^ (Pitafarm, Saratov, Russia), an anthelmintic injection containing the active substance Albendazole sulfoxide (Ricobendazole). It is a new-generation injectable drug and the first of its kind based on the active metabolite of Albendazole. The treatment is generally administered once a year in the second half of September as a preventive measure. Ricazole^®^ provides simultaneous action against all types of helminths, with an efficiency of 98–100%. It acts quickly, has high bioavailability, and effectively overcomes resistance to benzimidazoles.

The Ricazole^®^ was injected intramuscularly at 18 mL to each camel at a rate of 1 mL (4 mg) per 25 kg of body weight (according to the instructions for large ruminants: https://www.nita-farm.ru/produktsiya/rikazol/instruktsiya/, accessed on 28 November 2025). Indeed, according to the label, 1 mL contained 100 mg of ricobendazole, and the product provided 4 mg of active substance/kg body weight.

#### 2.2.2. Antiparasitic Drug

**Figure 1:**
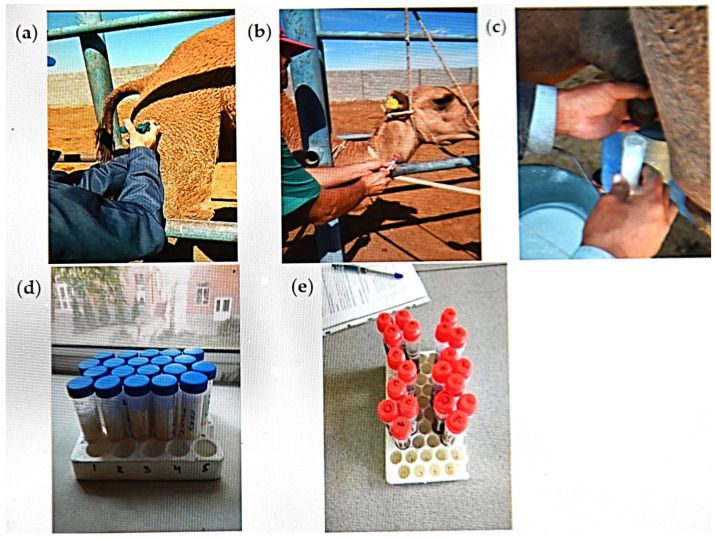
Photos from the course of the experiment: (**a**) intramuscular injection of the medication; (**b**) blood sampling; (**c**) milk collection during manual milking; (**d**) milk samples; (**e**) blood samples.

Ivermek^®^ (Pitafarm, Saratov, Russia), containing the active ingredient Ivermectin, is administered to animals for therapeutic and preventive purposes in cases of arachno-entomoses and nematodoses. It is an antiparasitic drug in injectable form, with treatment conducted once a year in the second half of September as a preventive measure. The drug provides simultaneous action against all types of parasites, ensuring fast effectiveness and high bioavailability. It was administered intramuscularly at a dose of 10 mL per camel (Figure 1), based on a dosage of 1 mL per 50 kg of body weight, following the instructions available at the following link: https://www.nita-farm.ru/produktsiya/rikazol/instruktsiya/ (accessed on 28 November 2025).

The dose and the date of the injections were decided with reference to the standard preventive deworming practice for the camel owners, where both drugs (Ivermectin and Albendazole) are administered once a year, generally the second half of September. A second injection is sometimes administered in the spring, before the grazing season. The weight of each camel ranged between 460 and 480 kg. No blood samples were taken in this group.

### 2.3. Chemicals and Reagents

Albendazole sulfoxide, VETRANAL reference standard (CAS No.54029-12-8, purity ≥ 99.0 %), was acquired from Merck KGaA, Darmstadt, Germany. Ivermectin, reference standard (CAS No. 70288-86-7, purity ≥ 99.0%), was purchased from Sigma Aldrich, Ltd. (St Louis, MO, USA). ACN was of HPLC grade and was provided by VWR, BDH Chemicals (Poole, Dorset, UK). Potassium phosphate and MeOH were of HPLC grade and were supplied by Sigma-Aldrich (St Louis, MO, USA), Ltd. The water (18.25 MΩ*cm, 25 °C) used in the experiment was purified and generated by an automatic water purification system (Direct-Q3 UV, Merck Drugs & Biotechnology Co., Inc., Fairfield, OH, USA). The solution entered into the HPLC system was first degassed by an ultrasonic apparatus (621.06.006, Isolab Laborgerate GmbH, Eschau, Germany).

### 2.4. Preparation of Standard Stock

The standard stock solutions of Albendazole sulfoxide were prepared individually at a concentration of 1 mg/mL by dissolving each target in ACN and then stored stably for two months in actinic glassware at −18 °C. Standard working solutions of 0.5 mg/mL, 0.1 mg/mL, and 0.05 mg/mL were prepared daily by gradually diluting the standard stock solutions with ACN.

The standard stock solutions of Ivermectin were individually prepared at a concentration of 1 mg/mL by dissolving each target in ACN + H_2_O (50:50) and were then stably stored for two months in amber glass containers at −18 °C. Standard working solutions of 0.5 mg/mL, 0.1 mg/mL, and 0.05 mg/mL were prepared daily by stepwise dilution of the stock solutions in ACN and were then stably stored for two months in amber glass containers at −18 °C. Standard working solutions of 0.5 mg/mL, 0.1 mg/mL, 0.05 mg/mL, and 0.01 mg/mL were prepared daily by stepwise dilution of the stock solutions in ACN.

### 2.5. Sample Acquisition and Preparation

#### 2.5.1. Sample Acquisition

Milk samples were taken before injection and were then taken 20 min (0.02) and 1, 2, 3, 4, 20, 34, 48, 62, 87, 101, 115, 130, 144, 158, and 172 days after injection. Milk was collected immediately into sterile 50 mL centrifuge tubes (40–45 mL × 2) during milking. Blood samples were taken from a blood vessel in the neck area directly into serological tubes. In case of hemolysis, which occurred especially when the weather was very cold, all blood samples from the same date were discarded. Thus, the agenda of blood sampling wasas follows: day 0 (before injection) and then 20 min (day 0.02) and 1, 2, 3, 4, 20, 62, 115, 144, and 172 days after injection. Blood samples were transported to the laboratory and kept warm for better coagulation. The milk samples were transported in refrigerated bags with coolants at a temperature of 5–7 °C. Then, they were immediately placed in a freezer at −20 °C.

Upon receipt of the blood samples in the laboratory, the blood serum was isolated into clean tubes and then stored at a temperature of −20 °C

#### 2.5.2. Sample Preparation

Sample preparation was performed according to Blanco-Paniagua et al. [16]: 200 µL of ethyl acetate was added to each 100 µL aliquot of milk and plasma. The mix was vortexed horizontally for 1 min and then centrifuged at 1200× *g* for 10 min at 4 °C. The supernatant was collected and evaporated to dryness under N2 at 30 °C. Measures of 500 µL of hexane and 300 µL of acetonitrile were added to evaporated samples, and the mix was vortexed horizontally for 1 min and then centrifuged at 1200× *g* for 10 min at 4 °C. Hexane was eliminated, and the rest was evaporated to dryness under N2 at 30 °C. Samples were resuspended in 100 µL of cold methanol and injected into the HPLC system. Samples from in vitro assays were injected directly into the HPLC system.

### 2.6. Instruments and Conditions LC

#### 2.6.1. Ricazole

The conditions for HPLC analysis of Albendazole sulfoxide were based on a method described by Blanco-Paniagua et al. [16], with modifications. The mobile phase used was potassium phosphate (pH 7)–acetonitrile (75:25) with a flow rate of 1.20 mL/min and a UV absorbance of 225 nm.

#### 2.6.2. Ivermectin

Chromatographic separation was performed on an LC-20 Prominence system (Shimadzu, Kyoto, Japan), with detection achieved using a UV detector (Shimadzu, Kyoto, Japan). The targets were retained using a BAKERBOND Q2100 Phenyl-Hexyl column (4.6 mm × 250 mm, 3 microns). Instrument connection and condition control were managed using LabSolutions software (Shimadzu, Kyoto, Japan). The column thermostat temperature was maintained at room temperature, and the injection volume was 20 µL. The mobile phase consisted of MeOH:ACN (1:1) + 42 mL H_2_O with a flow rate of 1 mL/min. The duration was 12 min, and the detection wavelength was set to 225 nm during this period.

#### 2.6.3. Quality Control

To ensure analytical reliability, blank camel milk samples—previously confirmed to contain no Albendazole or Ivermectin residues—were included in every analytical sequence. Their injection enabled the verification of the specificity, the absence of background contamination, and the stability of the chromatographic system. In addition, pure methanol was injected at regular intervals within each sequence to prevent possible cross-contamination between samples, as methanol was also used for reconstitution of extracts after evaporation.

Milk and blood samples were analysed in chronological order, starting from the pre-treatment (day 0) controls and continuing according to the sampling schedule after animal injection. This order reflected the expected decline in analyte concentrations over time and ensured a clean progression from low to higher concentrations, minimizing carry-over risk and maintaining consistency in the pharmacokinetic assessment.

### 2.7. Method Validation

For Albendazole- and Ivermectin-related residues, calibration was linear within the concentration range of 0.05–0.5 mg/mL. For the calculation of precision, LOD, and LOQ, a blank camel milk sample, free of target analytes, was used. This blank sample (*n* = 3) was spiked with Albendazole and Ivermectin at 0.05 mg/mL. Additionally, recovery rates (R) were determined.

To avoid potential ambiguity in unit interpretation, it is clarified that different units were intentionally applied at different analytical stages. Concentrations of stock and working standard solutions used for calibration are expressed in mg/mL, analyte concentrations in milk samples are reported in µg/mL, and LOD/LOQ values are presented in ng/L to reflect the lowest measurable levels. These unit conventions are applied consistently within each subsection.

The results of the validation parameters of the method are presented in Table 1.

### 2.8. Statistical Analyses

The mean and standard deviation were calculated using the “descriptive statistics” procedure of the software XLSTAT (Addinsoft ©, 2025, Paris, France).

To simultaneously compare the kinetics of the two molecules in the same graph (Albendazole and Ivermectin), we used index 100 at d0 for both of the molecules, calculated as follows: index value at d0 = X^0^/X^0^ × 100. Meanwhile, for the other days, the index values were X^n^/X^0^ × 100 with X^0^ = the concentration of Albendazole or Ivermectin at d0 and X^n^ = concentrations for all other d(n).

To compare the patterns of Albendazole and Ivermectin in milk as well as the patterns of Albendazole in milk and blood, we used the non-parametric Kolmogorov–Smirnov test (comparison of two distributions).

For all statistical analyses, the XLSTAT software, described above, was used.

## 3. Results

The water intake conditions were stable and are not expected to introduce any variability in pharmacokinetic parameters.

### 3.1. Albendazole Residues

**Figure 2:**
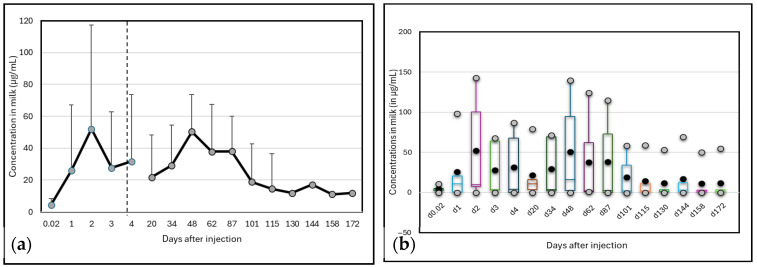
Changes in Albendazole concentrations (µg/mL) in camel milk after injection at day 0: (**a**) mean and standard deviation; (**b**) box plots representing mean, SD, min and max values.

High variability occurred between animals. Two camels did not excrete a high quantity of Albendazole molecules in milk, with a maximum concentration at around 20 µg/mL, while the three other camels in this group presented an important peak of excretion above 100 µg/mL. However, the peak of excretion appeared at different times: on day 2 for two camels and on day 48 for the last one. One of the camels which presented a peak at day 2 showed a second, less important peak (but still above 100 µg/mL) on day 87. Consequently, the mean kinetic (Figure 2) showed maximum concentrations on day 2 (52 ± 65.2 µg/mL) and day 48 (50 ± 63.1 µg/mL). After 172 days, the residues of Albendazole remained at around 11.9 ± 23.9 µg/mL, i.e., 2 to 5 times the concentration at day 0.

### 3.2. Ivermectin Residues

**Figure 3:**
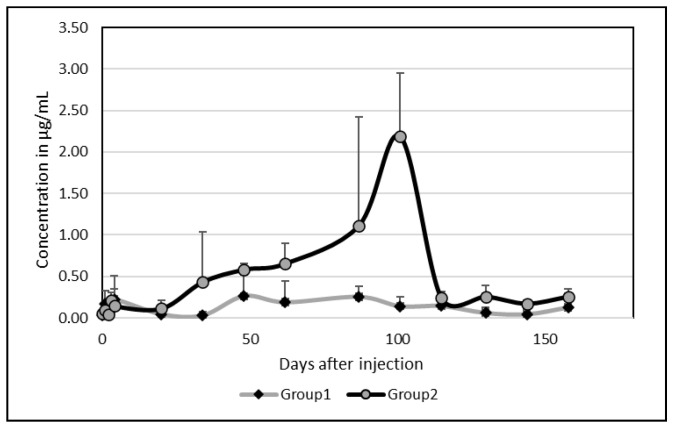
Changes in Ivermectin concentrations in camel milk in the two groups of camels without (group 1) and with (group 2) a peak of excretion.

As for Albendazole, there was an important between-camel variability. Among the five camels, two presented an important peak of excretion in milk at day 87 and 101 with values above 2 µg/mL (2.04 and 2.72 µg/mL, respectively). In the comparison of the kinetics between these two groups, without (group 1) and with (group 2) a peak of excretion, a clear difference was shown (Figure 3).

**Figure 4:**
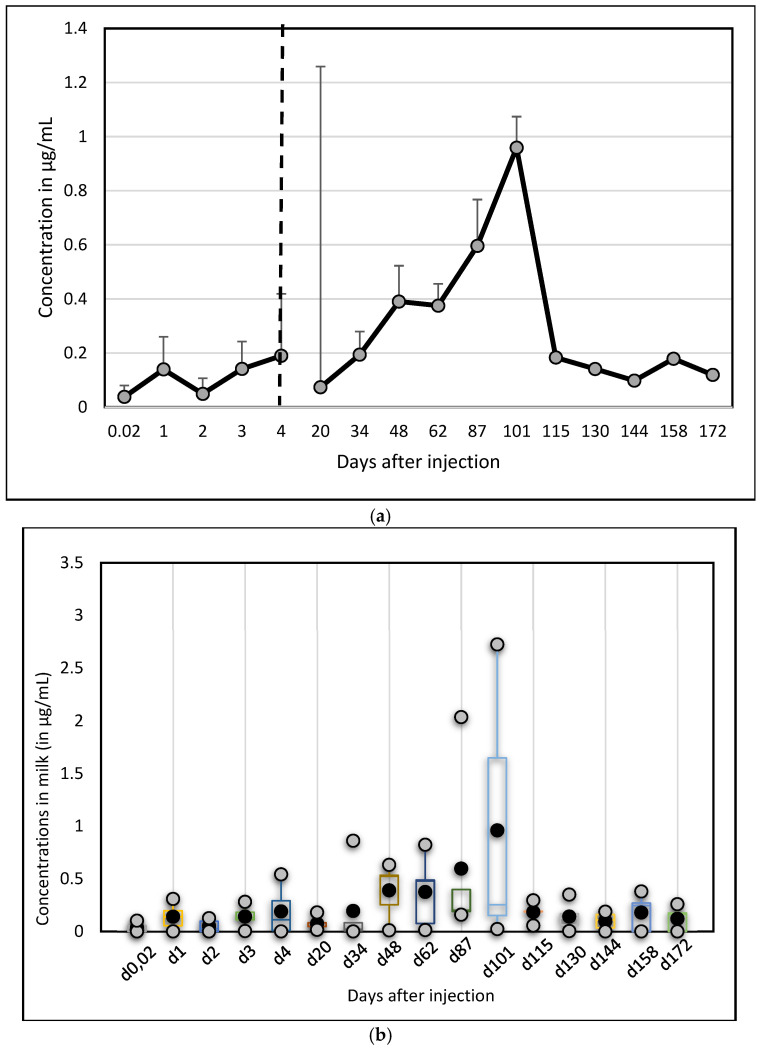
Changes in Ivermectin concentrations (µg/mL) in camel milk after injection at day 0: (**a**) mean and standard deviation; (**b**) box plots representing mean, SD, min and max values.

Contrary to Albendazole, however, on average, only one peak appeared on day 101 (0.96 ± 1.19 µg/mL). After 172 days, the mean concentration was still 0.12 µg/mL (Figure 4) but Ivermectin disappeared completely after 130 days in one camel and after 144 days in a second one.

### 3.3. Comparative Kinetics

**Figure 5:**
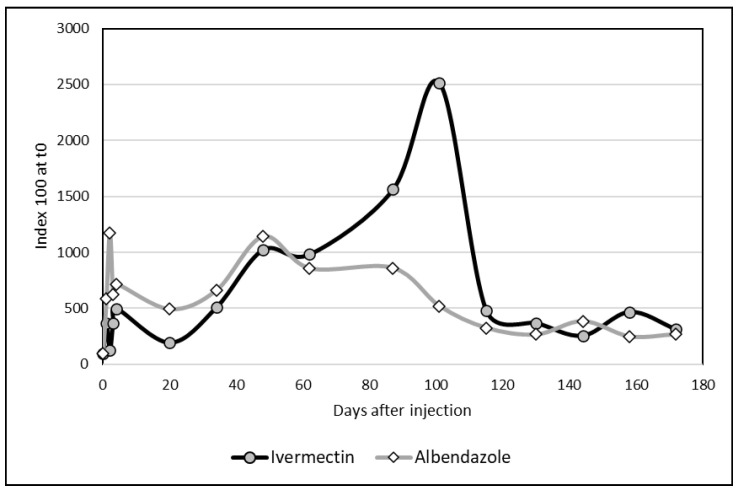
Comparative kinetics of Albendazole and Ivermectin residues in camel milk after injection (index 100 at d0).

The two graphs regarding Albendazole and Ivermectin could be compared by using index (100 at d0 for both molecules). If the kinetics appeared comparable until d48, the changes in Ivermectin differed importantly due to a sharp peak at day 101. However, the differences between the two distributions were not significant (Figure 5) according to the non-parametric Kolmogorov–Smirnov test.

**Figure 6:**
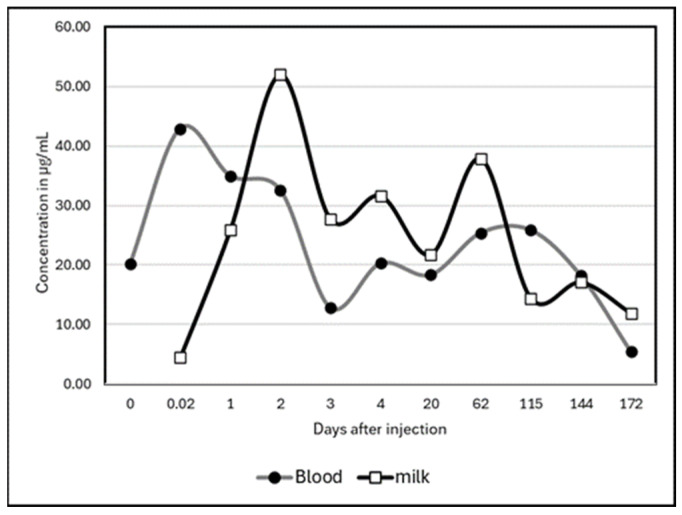
Comparative kinetics of Albendazole residues in blood and milk of camel after injection at d0.

Regarding the comparison of kinetics in blood and milk, only a study on Albendazole was available. The comparison was statistically non-significant (test of Kolmogorov–Smirnov); however, the two graphs showed a slight gap between blood concentrations and milk concentrations during the first days of the experiment (Figure 6).

## 4. Discussion

Antiparasitic residues in milk have been widely investigated in different farm animals [17] and in specific species, such as bovine [18,19,20], ovine [21], caprine [22,23,24], and donkey [25]. However, most of these investigations have been focused on the detection of residues in blood or milk samples, regardless of the delay from antiparasitic distribution and of the method of administration (injection, bolus, or tablets). The rare references for anthelmintic residues in camels are limited to their pharmacokinetic presence in plasma [26]. To our knowledge, no research regarding camel milk was available, except a few studies on the pharmacokinetics of Ivermectin [2] or similar molecules [10]. Moreover, comparisons with other species are difficult because the residues depend on the species, the administration method, and on the dose administered [27]. Another difficulty is due to the high variability in milk production which could impact the assessment of the drug concentrations because of the potential dilution effect. Such high between-camel variability was also observed in our study, although all the camels were at similar lactation stage and had comparable milk production. Indeed, due to the seasonal reproduction cycle in this species, all the camels were at the end of lactation (September) which is the month in which the anthelmintic treatment is generally administered at the entry of the autumn season. Our preliminary results are important, in that we can use them to suggest withdrawal periods for camels; currently, this period is based on recommendations regarding cattle, which appears to be a faulty approach [28,29]

### 4.1. Residues of Albendazole in Milk

Regarding Albendazole, the concentration in milk varied according to the metabolites of the molecules, notably Albendazole sulphoxide (ABZSO), Albendazole sulphone (ABZSO^2^), and Albendazole 2-aminosulphone (NH2ABZSO^2^). In their study in sheep milk, De Liguoro et al. [21] reported that the molecule of Albendazole was rapidly oxidized to ABZSO and then to ABZSO^2^ after administration and were present in milk at high levels (1–4 µg/g) for 24 h after treatment. Although reported values by Santos et al. [11] were 100 times higher than in other references, the highest Albendazole concentration observed in goat milk occurred 24 h after administration with values of 101.20 ± 37.4 μg/mL, and the molecule was not detectable in 50% of the samples after 72 h. After 4 days (96 h), Albendazole was not detectable in 100% of the samples. According to Moreno et al. [27], in cow milk, the maximal concentration was observed 12 h after administration; however, it was detected at a level around 0.28 µg/mL after administration by SC injection, while oral administration led to higher residues in milk, up to 1.12 µg/mL. In goat milk again, the maximum concentration of Albendazole metabolites were also observed on the first day after administration with a rapid decline until the fourth day, where the level of residue was below the MRL (0.1 µg/mL according to European Agency for the evaluation of Medicinal Products and Codex alimentarius: Source: “Residues of some veterinary drugs in foods and animals”—https://www.fao.org/4/y5612e/y5612e00.pdf, accessed on 28 November 2025), leading to recommend a withdrawal period of four days [24].

In a previous study including four cows receiving 10 mg/kg LW of Albendazole, Fletouris et al. [30] found higher concentrations of the metabolites after 12 h, but they reported different patterns according to the metabolites involved: Albendazole sulfone reached its maximum at 24 h (on average 0.81 µg/mL), while Albendazole sulfoxide was higher 12 h after administration (on average 0.52 µg/mL). The third metabolite (2-aminosulfone Albendazole) appeared in a lower quantity, with a peak at 36 h (0.13 µg/mL on average). In all the cases, a rapid elimination occurred, especially for sulfoxide and sulfone metabolites (below MRL after 48 h), and the third metabolite declined more slowly, reaching MRL after 3–4 days.

Higher sulfone metabolite concentrations in cow milk, 12 h after injection, were also reported recently by Imperiale and Lanusse [31], respectively, with 0.86 µg/mL for sulphone and 0.28 µg/mL for sulfoxide, followed by a rapid elimination after 24 h.

Compared to the other species, the pattern of Albendazole excretion in camel milk appeared quite different. It is notably marked by a slow decline, and, even if a peak was observed 48 h after injection, a second occurred on day 48, which has not been observed in other species. At the end of lactation, daily milk production can vary considerably, which might explain the anormal concentrations of residues.

The Albendazole residue in plasma appeared earlier than in milk in our study. Similar observations have been performed in sheep plasma for a long time [32], but with differences between the metabolites; here, Albendazole sulphoxide appears more rapidly (peak at 1.98 µg/mL less than 8 h after administration), while sulphone appears in plasma more slowly, with a maximum of 0.5 µg/mL 24 h after intraruminal administration. In an experiment in calves receiving Albendazole by intraluminal injection, the main metabolite (sulphone) appeared within 21 h after [33] with a peak between 1.89 and 2.53 µg/mL, depending on the type of diet. Sulphoxide metabolite also appeared sooner (between 11 and 18 h post-administration) depending on the diet. The plasma kinetics were similar between sheep and goat [34].

### 4.2. Residues of Ivermectin in Milk

The pattern of Ivermectin excretion in camel milk was quite different, with maximum excretion occurring a long time after administration; in our case, after more than 3 months. This was never observed in other species. Even if this peak was due to the exceptional maximum concentration in two camels out of five, the global Ivermectin residue remained important even up to 5 months (172 days) and above the MRL (0.01 µg/mL according to the Committee for Medicinal Products for Veterinary Use of European Union and Codex alimentarius: Source: “Residues of some veterinary drugs in foods and animals”—https://www.fao.org/4/y5612e/y5612e00.pdf, accessed on 28 November 2025).

In cattle treated with microdoses of Ivermectin, Alvinerie et al. [35] found a maximum in plasma concentration (0.46 ± 0.20 ng/mL) on the first day after administration, and in milk (0.27 ± 0.16 ng/mL) at day 3. In goat, the same authors [22] observed a slightly different pattern, with a maximum both in plasma (6.12 ng/mL) and milk (7.26 ng/mL) after 2.8 days with half-life absorption of 1.2 days. However, globally, there was high variability between the references and species, regarding milk, both for the values of the maximum residue (Cmax) and the time of the maximum (tmax). Thus, in cow milk, Cmax could vary between 0.27 and 75 ng/mL and tmax was between 1.8 and 8 days; in sheep milk, these values were 10–22.7 ng/mL and 1–1.3 days, and in goat milk, these values were 3–24 ng/mL and 1 to 3 days, respectively [24]. In Holstein cow milk, using ultra-high-performance liquid chromatography tandem–MS/MS analysis, Yang et al. [36] found Ivermectin residues even in cows treated within 10 d before calving, with a peak concentration in milk of 13.75 ± 8.12 ng/mL at 1.70 ± 1.68 d post-calving.

The only study involving camel milk is that of Oukessou et al. [2]. In their study, the authors found a Cmax of 2.74 ng/mL and a tmax of 17.3 days. Thus, even if our results differ from those of these authors, it seems that the Ivermectin molecule is excreted in camel milk later than it is in the other species, leading one to consider a longer withdrawal period in lactating camels. In a study regarding the comparative metabolism of enzymatic detoxification in different species (sheep, goat, cattle, and camel), the camel appeared less efficient, leading the authors of this paper to conclude that camels did not use the same enzymatic approach in the metabolism of the molecules as in medicine [37].

### 4.3. Risks for Consumers

Due to the lipophilicity of molecules like Ivermectin, even with doses below the MRL (0.01 µg/mL), the risk of chronic contamination may occur in consumers, and can have a deleterious effect on the consumers; this is especially the case when the molecules are detectable several days or even months after administration. Notably, Ivermectin can persist in goat and cow milk for 25 days and persists for 3 weeks in sheep milk; according to our observation, it remains for almost 6 months in camel milk.

The main risk for consumers is allergic reactions, such as skin rashes and respiratory issues, and endocrine and neurological disruptions [38]. Anthelminthic residues could also act on the immune systems of children, affecting their development and their health. Albendazole could even have a teratogenic effect, as detected in pregnant mice [39]. Long-term exposure to low doses could also contribute to drug resistance, affecting future antiparasitic treatments for consumers.

## 5. Conclusions

The preliminary results of this study, obtained with a limited number of animals, confirmed that applying the bovine-based withdrawal period to camel milk is not suitable. Indeed, in most of the studied camels, even after almost 3 months post-injection, the Albendazole and Ivermectin residues in the milk were still above the MRL. Thus, the regulations regarding the commercialization of camel milk cannot be based on the pharmacokinetics knowledge for antiparasitic treatments and prevention that have been obtained from other species. The persistence of the residues in camel milk, especially Ivermectin, appeared for longer, increasing the risk of exposure for regular consumers. Clear guidelines and more research on residue dynamics (including the administration method, the quantity of medicine administered, and the type of camel (Bactrian or dromedary)) are necessary to ensure consumer safety.

## Figures and Tables

**Table 1:** Linear regression equations, correlation coefficients (R^2^), CV (%), LODs, and LOQs of the HPLC-UV method for Albendazole and Ivermectin residues in camel milk.

Analyte	LRE	R^2^	CV, %	LOD, ng/L	LOQ, ng/L	R, %
Albendazole	y = 1 × 10^8^x	0.9995	0.2	0.31	0.93	99.2 ± 1.3
Ivermectin	y = 1.5687 × 10^4^x	0.9985	0.7	1384.2	4494.6	93 ± 1.9

LRE—linear regression equation; R^2^—correlation coefficient; LOD—limit of detection; LOQ—limit of quantification; R—recovery rate.

## Data Availability

The original contributions presented in this study are included in the article. Further inquiries can be directed to the corresponding author.

## Abbreviations

The following abbreviations are used in this manuscript:

ABZSO	Albendazole sulfoxide
ABZSO2	Albendazole sulphone
ALB	Albendazole
can	Acetonitryl
HPLC	High-Pressure Liquid Chromatography
IVM	Ivermectin
LOD	Limit of Detection
LOQ	Limit of Quantification
LRE	Linear Regression Equation
LW	Life Weight
MRL	Maximum Residue Limit
NH2ABZSO^2^	Albendazole 2-Aminosulphone
